# Efficacy of Multimodal Work‐Up of Head and Neck Squamous Cell Carcinoma Lymph Node Metastasis of Unknown Primary

**DOI:** 10.1002/hed.70147

**Published:** 2025-12-26

**Authors:** Robin W. Jansen, Roland M. Martens, Obaida Abdulrahman, Laura Peferoen, C. René Leemans, Gerben J. C. Zwezerijnen, Jan‐Jaap Hendrickx, Pim de Graaf

**Affiliations:** ^1^ Department of Otolaryngology‐Head and Neck Surgery Amsterdam UMC, Vrije Universiteit Amsterdam Amsterdam the Netherlands; ^2^ Department of Radiology and Nuclear Medicine Amsterdam UMC, Vrije Universiteit Amsterdam Amsterdam the Netherlands; ^3^ Cancer Center Amsterdam, Imaging and Biomarkers Amsterdam the Netherlands; ^4^ Amsterdam UMC VU University Medical Center Amsterdam the Netherlands; ^5^ Department of Pathology, Amsterdam UMC, Vrije Universiteit Amsterdam Amsterdam the Netherlands; ^6^ Cancer Center Amsterdam, Cancer Biology and Immunology Amsterdam the Netherlands

**Keywords:** ^18^F‐FDG PET, EUA, head & neck carcinoma unknown primary, head and neck squamous cell carcinoma, MRI, trans‐oral surgical techniques

## Abstract

**Background:**

Head and neck squamous cell carcinoma (HNSCC) often presents with cervical lymph node metastasis, with 1%–4% of cases presenting as cancer of unknown primary (CUP). CUP poses diagnostic and therapeutic challenges and is linked to poorer survival outcomes. Its incidence is expected to rise with the increasing prevalence of HPV‐positive HNSCC. This study evaluated the use of MRI, ^18^F‐FDG PET, examination under anesthesia (EUA), and TORS‐assisted tonsillectomy and tongue base mucosectomy (TORS‐TE/TBM) in the work‐up of CUP.

**Methods:**

This single‐center retrospective study included 79 patients with cytologically confirmed CUP (years 2019–2024). HPV‐positive (*n* = 51) and HPV‐negative (*n* = 28) cases were evaluated separately. Primary tumor detection rates for MRI, ^18^F‐FDG PET and EUA were calculated. For HPV‐positive tumors TORS‐TE/TBM was additionally evaluated for primary tumor detection.

**Results:**

In HPV‐positive cases MRI and ^18^F‐FDG PET had detection rates of 45% individually (respectively) and 53% combined. Of detected cases, 37% were identified by a single modality. Post‐hoc image review increased the detection rate to 63%. Subsequent imaging‐guided EUA had a detection rate of 68%. When TORS‐TE/TBM was performed after negative EUA, tumor detection occurred in 50% of cases. Multimodal work‐up resulted in an over‐all detection rate of 75%. In contrast, HPV‐negative cases had a lower over‐all multimodal detection rate of 39%.

**Conclusion:**

MRI and ^18^F‐FDG PET each play a pivotal and complimentary role for the detection of primary tumors in HNSCC CUP. Image‐guided EUA and, in selected cases, TORS‐TE/TBM further improve detection. A multimodal approach including expert imaging interpretation is recommended for optimal tumor identification and treatment planning.

## 1 Introduction

The global incidence of head and neck squamous cell carcinoma (HNSCC) is estimated at more than 800 000 newly diagnosed cases annually [1]. HNSCC frequently presents with cervical lymph node metastasis, which is often diagnosed by ultrasound‐guided fine needle aspiration. In an estimated 1% to 4% of cases, HNSCC initially presents with cervical lymph node metastasis without identification of the primary tumor location [2, 3]. An increasing amount of CUPs may be clinically expected, since CUPs are often human papillomavirus (HPV)‐related and HPV‐positive HNSCC has shown a marked rising incidence [4, 5, 6]. These cancers of unknown primary (CUP) pose significant diagnostic and treatment challenges. Treatment of HNSCC with CUP often involves (chemo)radiotherapy targeting the entire mucosal area where the primary tumor may be concealed. This leads to morbidities and considerable lower overall survival for these patients compared to those with detected HNSCC [7, 8]. Optimization of the detection of the primary tumor in patients presenting with cervical lymph node metastases is therefore vital.

Various diagnostic modalities are employed in the work‐up of patients presenting with cervical metastasis. Patients undergo physical examinations including nasopharyngoscopy and flexible laryngoscopy, and imaging generally includes both MRI and ^18^F‐FDG PET/CT [9]. CUPs are relatively frequently HPV‐positive and may involve small primary tumors located in the oropharynx, with extensive cervical metastases [10, 11]. The less frequent HPV‐negative cases do not necessarily originate in the oropharynx, and the much rarer is EBV‐positive CUP suggests a primary tumor in the nasopharynx. HPV‐status and EBV‐status of the neck metastasis can therefore guide the search for the primary tumor. Moreover, skin evaluation may be especially important for HPV‐negative cases, as skin would be another potential location of primary tumor within this group. Recent advancements in imaging technology, particularly the increased resolution of both and ^18^F‐FDG PET and MRI, and the introduction of MRI sequences of diffusion weighted imaging (DWI) and ultrafast dynamic contrast‐enhanced (DCE) MRI in head and neck oncologic imaging have significantly improved diagnostic accuracy in detecting the primary tumor in case of cervical lymph node metastasis [8]. Often, patients with CUP undergo an examination under general anesthesia (EUA), during which biopsies are taken from clinically suspicious areas or areas indicated by imaging. Narrow‐band imaging, which utilizes specific wavelengths of light, is frequently employed to enhance the detection of primary tumors [12]. However, despite physical examinations, imaging and EUA, the primary tumor remains occult in a significant proportion of these patients [8, 13, 14, 15].

In the last two decades, the development of transoral robotic surgery (TORS) has expanded the diagnostic evaluation of cervical CUPs. For CUPs, TORS is useful as a tool for TORS‐assisted ipsilateral tonsillectomy and ipsilateral tongue base mucosectomy (TORS‐TE/TBM). Generally, this is performed as the fourth diagnostic tool after negative findings from MRI, ^18^F‐FDG PET, and EUA. TORS has become increasingly available in more medical centers. TORS‐TE/TBM is considered to improve detection rates of occult tumors, especially in HPV‐positive tumors, reducing the need for extensive radiation therapy and its associated side effects [16, 17, 18, 19, 20]. For HPV‐negative tumors, the added value of TORS‐TE/TBM in detecting the primary tumor is considerably limited. However, the decision to perform diagnostic TORS‐TE/TBM varies between centers [21].

The primary aim of this study is to evaluate the detection rates of MRI, ^18^F‐FDG PET, EUA, TORS‐TE/TBM, and the combination of these modalities in identifying the primary tumor in patients with lymph node metastasis in HNSCC. Furthermore, the study aims to evaluate the sensitivity and specificity of MRI and ^18^F‐FDG PET compared to histopathology.

## 2 Materials and Methods

### 2.1 Patient Selection

The Medical Ethics Review Committee of Amsterdam UMC approved this study with a waiver of informed consent (IRB00002991, FWA00017598). Patients with suspected head and neck tumors who presented at a tertiary head and neck oncology referral center (Amsterdam UMC) were reviewed between January 1, 2019, and May 1, 2024. Inclusion criteria were: cytologically confirmed cervical lymph node metastasis of squamous cell carcinoma (SCC) with a clinically unidentified primary tumor, following flexible laryngoscopy but prior to any imaging techniques (e.g., MRI and/or ^18^F‐FDG PET) or surgical interventions (e.g., EUA and/or TORS‐TE/TBM). Patients with a history of HNSCC were excluded.

### 2.2 Clinical Data

After including patients, a chart review was conducted to perform data collection, including information on patient demographics (age, sex), staging information (TNM), number and levels of malignant cervical nodes, HPV status, imaging results (MRI, ^18^F‐FDG PET), diagnostic surgical intervention outcomes (EUA, TORS‐TE/TBM), and pathology reports. Information about treatment with radiotherapy and the occurrence of recurrence or metastasis was also collected.

### 2.3 Diagnostic Modality Specifications

The standard sequence of diagnostic work‐up was MRI, ^18^F‐FDG PET and EUA. In HPV‐positive cases where no primary tumor was identified through diagnostic imaging or EUA, TORS‐TE/TBM was performed. MRI acquisition was performed on a 3T Magnetom VIDA (Siemens, Erlangen, Germany) using a 16‐channel neurovascular coil. Axial T1‐weighted (T1w) and axial STIR images were obtained. Diffusion‐weighted imaging (DWI) was conducted with two or three *b* values (0, 500, 1000 or 0, 1000 s/mm^2^). Read‐out segmented EPI (RESOLVE) had the following parameters: TR 2000, TE 56, slice thickness 4 mm, intersection gap 4 mm, matrix 150 × 150, field of view 219 × 219 mm, 2 averages. Fat‐suppressed spin‐echo echo‐planar imaging (SE‐EPI) had the following parameters: TR 8400, TE 55, slice thickness 4 mm, intersection gap 4 mm, matrix 176 × 256, field of view 219 × 219, 3 averages. The ADC map was generated using manufacturer‐provided software. Ultrafast DCE imaging was performed using Dotarem contrast with the following parameters: TR 540 ms, TE 1.92 ms, and 1 average. Both 3D and 2D acquisitions were used, with a slice thickness of 3 mm and an intersection gap of 4 mm. The field of view (FOV) was 220 mm × 220 mm, and the matrix size was 224 × 224. PET/CT images were acquired using optimized head and neck parameters, including 4 iterations, 16 subsets, and a 5 mm 3D Gaussian filter, with photon attenuation correction applied. The image matrix size was 144 × 144 with a voxel size of 4 × 4 × 4 mm. Low‐dose non‐contrast enhanced CT was performed with 50 mAs and 120 kV for anatomical correlation and attenuation correction, using a 512 × 512 matrix size, resulting in a pixel size of 1.17 × 1.17 mm and a slice thickness of 5 mm.

EUA was performed in the operating theater through visual examination of all mucosal surfaces of the oral cavity, pharynx, larynx, proximal esophagus, and trachea. Narrow‐band imaging (NBI) was utilized during the examinations. Biopsies were taken from clinically suspicious sites, radiologically suspicious sites, or areas of asymmetry or irregularity. No ‘blind biopsies’ were taken in the absence of clinical or radiological abnormalities.

TORS‐TE/TBM was performed in the operating theater using the Da Vinci (Si/Xi) robot to assess and perform mucosectomy of the base of the tongue. In our center, only HPV‐positive cases underwent TORS‐TE/TBM, a procedure that combines tonsillectomy with tongue base mucosectomy. It is typically performed as unilateral tonsillectomy combined with unilateral tongue base mucosectomy with extension just over the contralateral midline. Procedures were performed without the use of fresh frozen biopsies.

Surgical resection specimens were anatomically oriented by the operating surgeon, and resection margins were inked accordingly. The specimens were serially sectioned at 3 mm intervals and entirely embedded for histopathological evaluation. Microscopic examination was conducted by a dedicated head and neck pathologist. In cases where no tumor was identified on hematoxylin and eosin (H&E) staining alone, additional immunohistochemical staining with p16 and pan‐keratin was performed on all tissue sections to enhance the detection of small, poorly differentiated HPV‐related tumors.

### 2.4 Analysis

Radiology reports from MRI and ^18^F‐FDG PET scans were reviewed and assessed for suspected lesions. All radiology evaluations were performed by dedicated head and neck radiologists (experience range 15–26 years). Only histopathology‐proven detected cases were considered for calculating detection rates. Detection rates for separate and combined modalities were analyzed, stratified by HPV status. In patients when there was a discrepancy between the different modalities (MRI, ^18^F‐FDG PET, EUA, TORS‐TE/TBM), images were re‐evaluated (post hoc) by a radiologist (P.G.) and a nuclear medicine physician (G.Z.) with respectively 18 and 10 years of experience in head and neck imaging. Both reviewers were blinded to the final diagnosis, the clinical data, and the original imaging reports. The re‐evaluation involved a thorough assessment of both anatomical and functional sequences (Ultrafast DCE and DWI), with particular emphasis on asymmetry and abnormal dynamic contrast enhancement patterns, including very early focal mucosal enhancement and/or focal restricted diffusion. A sub‐analysis was conducted with the new data after re‐evaluating the MRI and ^18^F‐FDG PET images. Next to detection rates, sensitivity and specificity of MRI and ^18^F‐FDG PET were evaluated with pathology reports from EUA and TORS‐TE/TBM as gold standard. Statistical analyses in this study were performed using IBM SPSS Statistics version 28.0.0.1.

## 3 Results

### 3.1 Patient Characteristics

In total, 79 patients were included in this study (19 female, 24%). Of these, 51 (65%) were HPV‐positive and 28 (35%) HPV‐negative. Results are reported separately for HPV‐positive and negative patients. The median age was 63 years (range 42–89 years). Patient characteristics are detailed in Table 1. MRI and ^18^F‐FDG PET were performed on average 5.6 and 6.9 days after first consultation respectively. Pathology specimens were collected through EUA and TORS, on average 11.3 and 32.8 days after first consultation respectively.

**TABLE 1:** Patient characteristics.

Patient characteristics	HPV+ (*n* = 51)	HPV− (*n* = 28)	All (*n* = 79)
Median age (range)	61 (42–78)	67 (49–89)	63 (42–89)
Gender, *n* (%)			
Male	38 (75%)	22 (79%)	60 (76%)
Female	13 (25%)	6 (21%)	19 (24%)
Identified primary tumor (%)	38 (75%)	11 (39%)	49 (62%)
Primary tumor location, (%)			
Tonsil	18 (35%)	3 (11%)	22 (28%)
Base of tongue	18 (35%)	2 (7%)	19 (24%)
Tonsil and base of tongue	2 (4%)	0	2 (3%)
Hypopharynx	0	3 (11%)	3 (4%)
Larynx	0	2 (7%)	2 (3%)
Floor of the mouth	0	1 (4%)	1 (1%)
Unknown	13 (25%)	17 (61%)	30 (38%)
T‐stage, *n* (%)			
Tx	13 (25%)	17 (61%)	30 (38%)
T1	28 (55%)	10 (36%)	38 (48%)
T2	10 (20%)	1 (4%)	11 (14%)
N‐stage, *n* (%)			
N1	42 (82%)	4 (14%)	46 (58%)
N2	7 (14%)	0	7 (9%)
N2a	0	4 (14%)	4 (5%)
N2b	0	6 (21%)	6 (8%)
N2c	0	1 (4%)	1 (1%)
N3	2 (4%)	0	2 (3%)
N3b	0	13 (46%)	13 (16%)
M‐stage, *n* (%)			
M1	0	1 (4%)	1 (1%)
Performed diagnostic modality			
MRI	51 (100%)	27 (96%)	78 (99%)
^18^F‐FDG PET	47 (92%)	28 (100%)	75 (95%)
EUA	44 (86%)	28 (100%)	72 (91%)
TORS‐TE/TBM	16 (31%)	NA	NA

*Note:* The 8th Edition of the TNM Staging Manual, published by the American Joint Committee on Cancer (AJCC), was used for determining T‐, N‐, and M‐stage.

### 3.2 HPV‐Positive CUP: Primary Tumor Detection Rates

**FIGURE 1:**
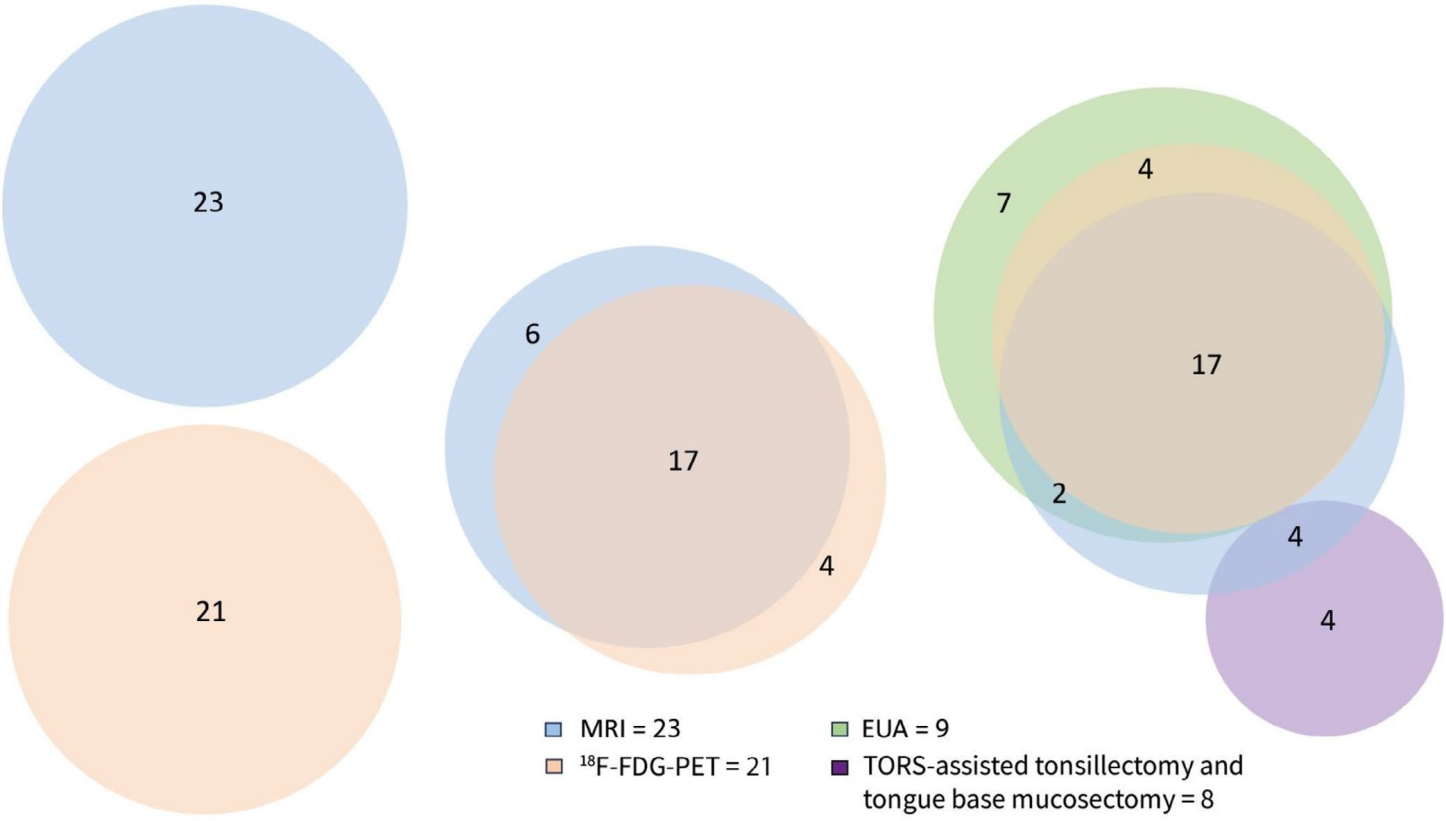
Venn diagram showing the codetection of the primary tumor by the different modalities for HPV‐positive tumors. [Color figure can be viewed at wileyonlinelibrary.com]

The detection rate for HPV‐positive CUP of all combined modalities was 75%, leaving 25% of primary tumors undetected after full diagnostic work‐up (Table 2). The detection rate for imaging modalities was 45% for MRI and 45% for ^18^F‐FDG PET. Imaging modalities combined detected 53% of primary tumors. When performing post hoc review of images, the detection rate retrospectively increased to 63% for imaging. Informed by initial imaging status, the detection rate for EUA was 68%. TORS‐TE/TBM yielded a detection rate of 50% in the group of 16 HPV‐positive patients that had no tumor detected on imaging or EUA. In the majority of cases that underwent TORS‐TE/TBM, bilateral mucosectomy combined with bilateral tonsillectomy was avoided (14/16, 88%). Although different modalities showed considerable overlap in the detection of primary tumors, the Venn diagram shows the added benefit of each individual modality in detecting the primary tumor (Figure 1). There was overlap between modalities, with the most overlap between MRI and ^18^F‐FDG PET in 17 out of 27 cases (63%) with still 37% being detected by one modality exclusively (Figure 1). Of eight patients with detected primary tumor through TORS‐TE/TBM, 6 (75%) were detected in the base of the tongue and 2 (25%) in the tonsil. In four cases, there were suspected lesions on imaging which were not confirmed on EUA with biopsies but were confirmed on subsequent TORS‐TE/TBM.

**TABLE 2:** Neck metastasis of the unknown primary: detection rate for primary tumors for each diagnostic modality (histopathology‐confirmed) stratified by HPV status.

Modality	HPV‐positive (*n* = 51)		HPV‐negative (*n* = 28)	
	Detection rate (detected cases/modality performed)	Detection rate post hoc^a^ (detected cases/modality performed)	Detection rate (detected cases/modality performed)	Detection rate post hoc^a^ (detected cases/modality performed)
MRI	45% (23/51)	59% (30/51)	22% (6/27)	26% (7/27)
^18^F‐FDG PET	45% (21/47)	55% (26/47)	21% (6/28)	25% (7/28)
EUA	68% (30/44)	NA	39% (11/28)	NA
TORS‐TE/TBM	50% (8/16)	NA	NA	NA
Combined modalities, detected on either:				
MRI or ^18^F‐FDG PET	53% (27/51)	63% (32/51)	25% (7/28)	29% (8/28)
MRI, ^18^F‐FDG PET or EUA	67% (34/51)	NA	39% (11/28)	NA
MRI, ^18^F‐FDG PET, EUA, or TORS‐TE/TBM	75% (38/51)	NA	NA	NA

*Note:* If denominators do not add up to 51 in HPV‐positive cases and 28 in HPV‐negative cases, the modality was deployed in not all patients (see Table 1).

^a^ Post hoc analysis: In patients, when there was a discrepancy between the different modalities (MRI, PET, EUA, TORS‐TE/TBM), images were re‐evaluated by a radiologist and a nuclear medicine physician.

### 3.3 HPV‐Positive CUP: Sensitivity and Specificity of Imaging Findings Compared to Histopathology

When comparing positive imaging findings with histopathology results, MRI had a sensitivity of 63% and a specificity of 39%. ^18^F‐FDG PET showed a comparable sensitivity of 60% and a specificity of 42% for HPV‐positive tumors. In blinded post hoc analysis, sensitivity was considerably higher for MRI (79% vs. 63%) and ^18^F‐FDG PET (74% vs. 60%) (Table 3).

**TABLE 3:** Sensitivity and specificity for imaging modalities for detection of the primary tumor with histopathology as gold standard.

HPV status	Modality^a^	Sensitivity compared to histopathology^b^		Specificity compared to histopathology^b^		Positive predictive value compared to histopathology^b^		Negative predictive value compared to histopathology^b^	
		Initial	Post hoc^c^	Initial	Post hoc	Initial	Post hoc	Initial	Post hoc
Positive (*n* = 51)	MRI	63%	79%	39%	31%	75%	77%	26%	33%
	^18^F‐FDG PET	60%	74%	42%	25%	75%	74%	26%	25%
	MRI and ^18^F‐FDG PET	49%	69%	100%	33%	100%	75%	40%	27%
	MRI or ^18^F‐FDG PET	74%	84%	42%	50%	79%	76%	36%	63%
Negative (*n* = 28)	MRI	50%	64%	60%	44%	46%	44%	64%	64%
	^18^F‐FDG PET	50%	64%	50%	47%	39%	44%	62%	67%
	MRI and	45%	55%	100%	44%	100%	40%	73%	58%
	MRI or ^18^F‐FDG PET	60%	73%	60%	47%	50%	47%	69%	73%

^a^ “MRI or ^18^F‐FDG PET” denotes histopathology confirmed positive finding on either imaging modality while “MRI and ^18^F‐FDG PET” denotes histopathology confirmed positive finding on the same location on both imaging modalities.

^b^ Sensitivity and specificity were calculated compared to histopathology acquired through EUA or TORS‐TE/TBM, considering negative imaging combined with a biopsy or TORS‐TE/TBM without malignancy a true negative. Of all patients, histopathology material was available (*n* = 51 for HPV‐positive and *n* = 28 for HPV‐negative cases).

^c^ Post hoc analysis: In patients when there was a discrepancy between the different modalities (MRI, PET, EUA, TORS‐TE/TBM), images were re‐evaluated by a radiologist and a nuclear medicine physician blinded to the final diagnosis, the clinical data, and the original imaging reports.

### 3.4 HPV‐Negative CUP: Primary Tumor Detection Rates

**FIGURE 2:**
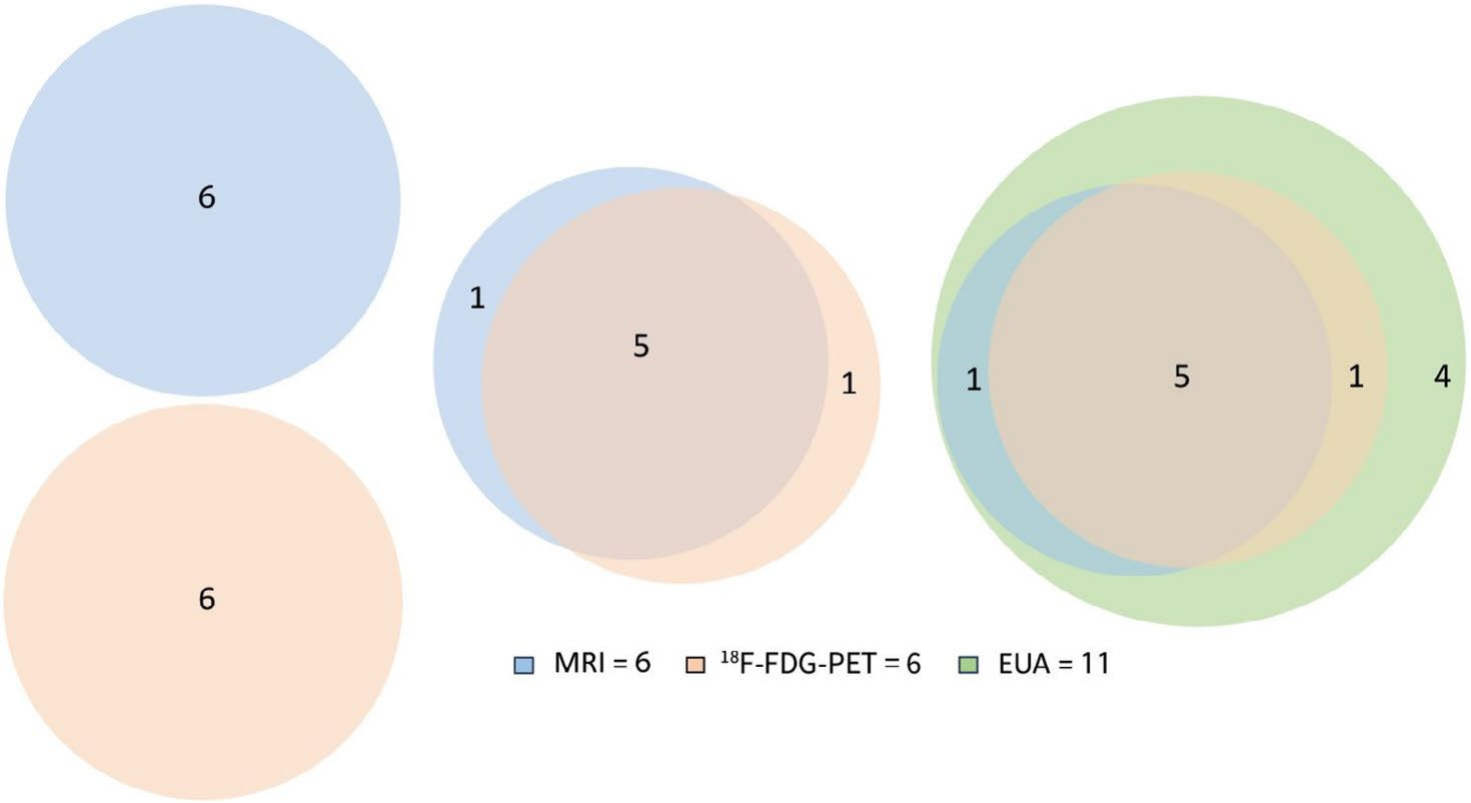
Venn diagram showing the codetection of the primary tumor by the different modalities for HPV‐negative tumors. [Color figure can be viewed at wileyonlinelibrary.com]

The detection rate for HPV‐negative CUP of all combined modalities was 39%, leaving 61% of primary tumors undetected after full diagnostic work‐up (Table 2). For HPV‐negative CUP, the detection rate for imaging modalities was 22% for MRI and 21% for ^18^F‐FDG PET. Imaging modalities combined detected 25% of primary tumors. When performing post hoc review of images, the detection rate retrospectively increased to 29% for imaging. Informed by initial imaging status, the detection rate for EUA was 39%. No diagnostic TORS‐TE/TBM was performed for HPV‐ CUP. Of the 11 detected primary tumors, 4 (36%) were exclusively detected by EUA (Figure 2).

### 3.5 HPV‐Negative CUP: Sensitivity and Specificity of Imaging Findings Compared to Histopathology

**FIGURE 3:**
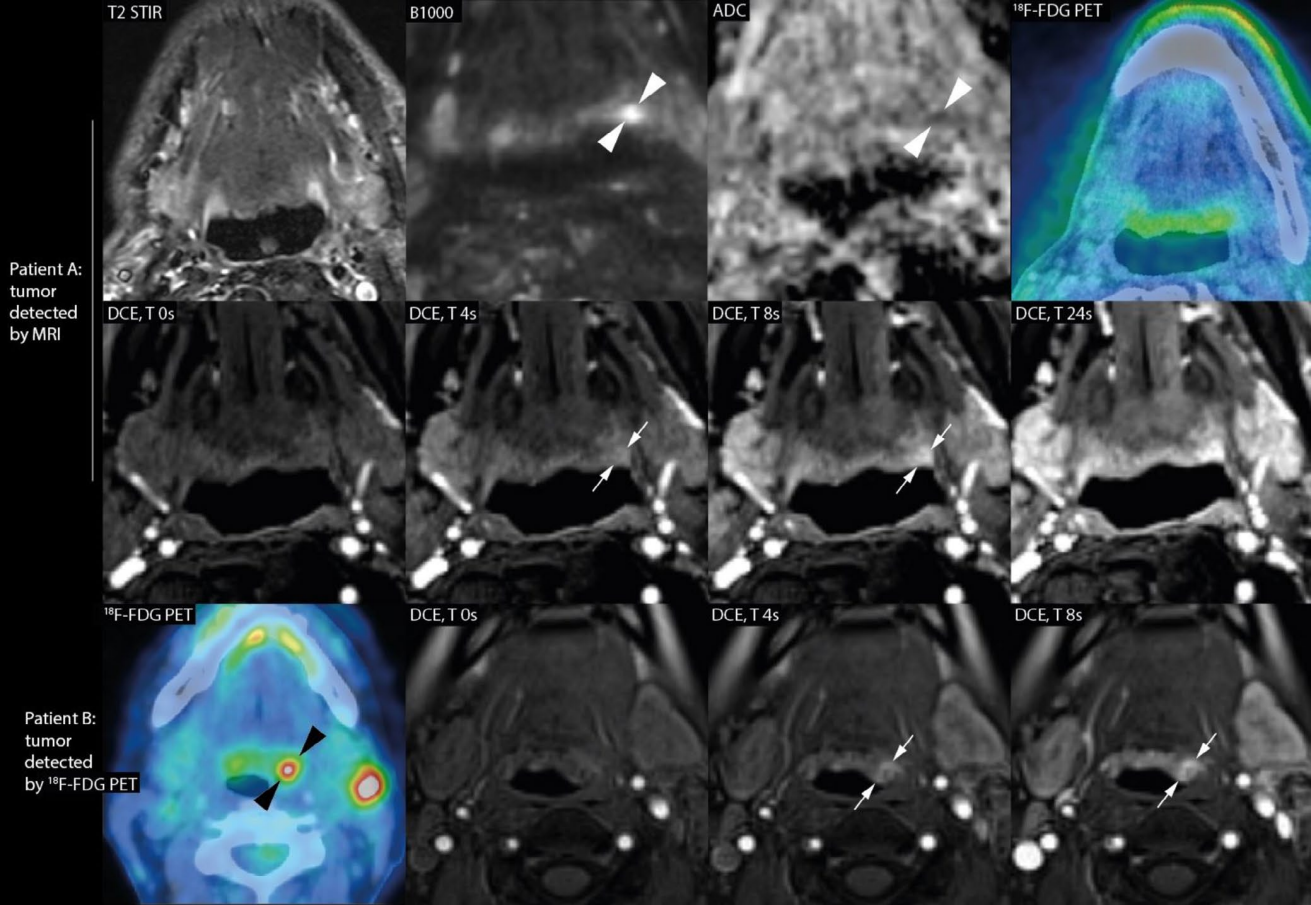
Individual benefit of separate diagnostic imaging modalities. Patient A: A patient presented with a neck metastasis in a left level 2 lymph node (HPV‐positive), where the primary tumor was detected exclusively by MRI, showing diffusion restriction at the tongue base (indicated by white arrowheads) and a corresponding asymmetric early enhancing mass on DCE (white arrows). The final staging was T1N1M0 tongue base carcinoma (histopathologically confirmed), treated with TORS‐assisted ipsilateral tonsillectomy and ipsilateral tongue base mucosectomy (TORS‐TE/TBM) followed by adjuvant chemotherapy and radiotherapy. Patient B: A patient presented with a neck metastasis in the left level 2 lymph node (HPV‐positive), where the primary tumor was initially identified only by ^18^F‐FDG PET (indicated by the black arrowhead). A post hoc analysis of the current study revealed a corresponding asymmetric early enhancing mass at the left tongue base on DCE, suggestive of the primary tumor (white arrows). The final staging was T1N1M0 tongue base carcinoma (histopathology confirmed), treated with chemotherapy and radiotherapy. [Color figure can be viewed at wileyonlinelibrary.com]

**FIGURE 4:**
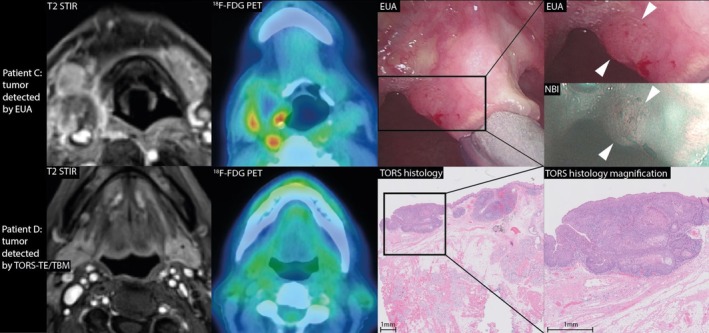
Individual benefit of separate diagnostic surgical modalities. Patient C: A patient presented with a neck metastasis in a right level 3 lymph node (HPV‐negative), where the primary tumor was detected exclusively through examination under anesthesia (EUA) at the lingual side of the epiglottis (indicated by white arrowhead). Irregular mucosa was directly visible, and narrow‐band imaging (NBI) enabled visualization of a neoangiogenic pattern of mucosal vasculature. Although the ^18^F‐FDG PET does show FDG uptake of the neck metastasis, the primary tumor is undetected on both ^18^F‐FDG PET and MRI. The final staging was T1N3bM0 carcinoma of the epiglottis (histopathology confirmed), which was treated with radiotherapy. Patient D: A patient presented with a neck metastasis in a left level 2 lymph node (HPV‐positive), where the primary tumor was detected exclusively through TORS‐assisted ipsilateral tonsillectomy and ipsilateral tongue base mucosectomy (TORS‐TE/TBM). The final staging was pT1N1 left tongue base carcinoma (histopathology confirmed), which was treated by TORS‐TE/TBM and neck dissection (levels II–IV), without adjuvant treatment. [Color figure can be viewed at wileyonlinelibrary.com]

When comparing positive imaging findings with histopathology results, MRI had a sensitivity of 50% and a specificity of 60% in HPV‐ CUP. ^18^F‐FDG PET showed a comparable sensitivity of 50% and a specificity of 50%. In blinded post hoc analysis, sensitivity was considerably higher for MRI (64% vs. 50%) and ^18^F‐FDG PET (64% vs. 50%) (Table 3). Figures 3 and 4 show examples of the individual benefit of the separate diagnostic modalities, both imaging modalities (Figure 3) and surgical modalities (Figure 4).

## 4 Discussion

Neck metastasis with an unknown primary tumor poses a significant clinical diagnostic challenge, as failure to detect the primary tumor is associated with worse prognosis [22]. The anticipated increase in the incidence of CUP due to the rise of HPV‐associated HNSCC underscores the growing urgency of research in this field. Detection rates for HPV‐positive tumors (75%) were considerably higher than for HPV‐negative tumors (39%), leaving, however, a substantial proportion of primary tumors undetected. The improved detection rates achieved with the use of both MRI and ^18^F‐FDG PET underscore the importance of incorporating both imaging modalities into the standard diagnostic work‐up for CUPs [23, 24]. Incorporating surgical interventions such as imaging‐informed EUA with NBI and TORS‐TE/TBM further increased primary tumor detection rates and has both been shown to be essential for optimized CUP diagnostics [19, 24].

Diagnostic performance of imaging was comparable to that in recent literature, and results support the use of both MRI and ^18^F‐FDG PET. This study evaluated both detection rate (detected cases/total cases) and sensitivity (detected cases by imaging/detected cases by pathology). The detection rate for MRI was comparable to other studies for HPV‐positive cases, with a rate of 45% compared to 40%–41% in other studies [23, 24, 25, 26]. Similarly, the detection rate for 18F‐FDG PET was also consistent with other studies, showing a rate of 45%, which aligns with the 45%–69% range previously reported [23, 24, 25, 26]. When comparing imaging results to pathology findings, the initial sensitivity in this study was lower compared with literature (81% for MRI and 93% for PET‐CT [27]), but re‐evaluation of images brought sensitivity to levels more comparable with previous studies. Therefore, the discrepancy may be attributed to the fact that previous imaging studies used re‐evaluations of images instead of clinical reports for analysis. Different cohorts of patients with CUP may however not be comparable due to their heterogeneity in terms of patients' characteristics, HPV‐status, regional epidemiology, healthcare delay, and type of original work‐up (e.g., NBI at initial laryngoscopy). The added benefit of individual modalities however remains consistent. A considerable amount of cases were detected by a single imaging modality (MRI or ^18^F‐FDG PET), suggesting the use of both modalities simultaneously before conducting EUA in order to optimize detection rates and expedite the diagnostic work‐up [23, 24].

Improved primary tumor detection after re‐evaluations suggests that double reading could be beneficial in cases where the primary tumor remains occult after first reading. The frequent observation of early enhancement and washout on DCE‐MRI at the site of the primary tumor suggests that careful integration of these sequences is essential. In evaluating images, it is recommended to aim for maximal sensitivity in reading since false‐positive imaging findings only have minor consequences.

Both MRI and ^18^F‐FDG PET showed higher detection rates in HPV‐positive cases compared to HPV‐negative cases, although each modality did show added benefit in both groups. The increased detection for HPV‐positive tumors may be attributed to the fact that imaging review can be targeted at the oropharynx with tumors most found in the palatine tonsil and base of the tongue [8, 28]. For MRI, HPV‐positive tumors show more pronounced restricted diffusion on DWI‐MRI and often grow expansively rather than infiltratively [29, 30]. MRI can however be hindered by both movement‐ and dental artifacts. In the clinical setting, MRI is often favored over CT because of the higher sensitivity [23]. For ^18^F‐FDG PET, HPV‐positive tumors are known to have increased glycolytic activity [31]. ^18^F‐FDG PET may however have limitations of insufficient resolution for small lesions (< 5 mm), physiological tonsil uptake, and glucose metabolism changes in diabetic patients [8, 32, 33]. Moreover, on ^18^F‐FDG PET, it may be hard in cases to distinguish between lymph nodes and the primary tumor, if located in close proximity.

EUA generally follows after imaging, making the technique guided thereby potentially overestimating its intrinsic value. However, histopathological confirmation of suspected lesions is vital before initiating treatment. EUA may be especially important in HPV‐negative cases where the diagnostic capabilities of imaging are limited. In our study, EUA showed comparable detection rates to those in other studies [34, 35]. It is advised to biopsy every suspicious area, nodularity, or roughness in the mucosa. The implementation of NBI in the outpatient clinic or during the EUA is recommended as it has shown added value through higher detection rates [12].

TORS‐TE/TBM was deployed in a subset of patients, generally as the last step after other diagnostic modalities in the work‐up of HPV‐positive tumors [36]. Tonsillar and tongue base crypts may harbor small tumors which can be difficult to detect through imaging or EUA, potentially explaining the relatively high rate of cases exclusively detected through TORS‐TE/TBM [14]. TORS‐TE/TBM detected tumors were more frequently located at the base of the tongue rather than the tonsil, consistent with previous literature [19, 37]. The lower primary tumor detection rate for TORS‐TE/TBM (50%) compared to a recent systematic literature review (72%) is potentially explained by the difference in pre‐TORS‐TE/TBM diagnostic work‐up which included DWI‐MRI and ultrafast DCE‐MRI in the current study, which was lacking in various studies in the systematic review [19]. The added benefit of TORS‐TE/TBM may generally outweigh negative aspects including increased costs, transient post‐operative pain and dysphagia, and minor risk of bleeding [19, 21, 38]. Both the ability to assess suspicious areas under magnification and the ability to perform a resection of the area at risk as extensively as possible offers significant advantages of TORS‐TE/TBM [39, 40]. In some cases with small primary tumors, TORS‐TE/TBM may serve as a therapeutic tool to excise the primary tumor, potentially offering the substantial advantage of a monomodal therapy (surgery‐only) preventing radiation toxicity, provided that tumor‐free margins are achieved [41, 42, 43]. Within this study only HPV‐positive patients underwent TORS‐TE/TBM; therefore, no conclusions can be drawn on the use of TORS‐TE/TBM for HPV‐negative cases. The relatively high detection rate of TORS‐TE/TBM in HPV‐positive tumors, however, does support the use of it in the diagnostic work‐up of HPV‐positive tumors.

Different explanations exist for a primary tumor remaining undetected after EUA and/or TORS‐TE/TBM. Naturally, this may be due to techniques failing to identify the tumor. Alternatively, the immune clearance at the primary tumor site may play a role. In case of clearance, suspected lesions on initial imaging may have represented the primary tumor, even if consequent histopathology results are negative. Although immune clearance is a known phenomenon, its rate is difficult to estimate. It is unclear why clearance would occur at the primary tumor site, but is not effective in the draining lymph nodes. This could imply alterations in the tumor cell behavior or phenotype at the metastatic site, such as downregulation of tumor antigens or major histocompatibility complex (MHC) molecules or induction of immune suppressive modulators such as suppressive cytokines (e.g., interleukin‐10, transforming growth factor‐beta) or immune checkpoint ligands (e.g., B7‐H molecules like PD‐L1, PD‐L2, B7H3) [44, 45]. HPV‐positive tumors have shown more immunogenicity compared with HPV‐negative tumors [44, 45, 46]. Potential other hypotheses for not finding the primary tumor may involve the presence of small submucosal lesions with slow growth rates or the early occurrence of mutations that promote lymphatic migration, such as overexpression on SEC62 through 3q26 chromosomal amplification [47].

This study had several limitations. Firstly, the retrospective nature of the study introduces potential biases, such as selection and information bias. The lack of a standardized prospective setting introduced heterogeneity regarding diagnostic work‐up for example, for timing and order of different modalities. The use of data from a single center limits the generalizability of the findings. Finally, histopathology was not re‐evaluated, which could have enhanced the accuracy and consistency of the diagnoses.

## 5 Conclusion

Both MRI and ^18^F‐FDG PET have individual value for detecting the primary tumor in both HPV‐positive and HPV‐negative CUP. In case of initial negative imaging results, a second reader may enhance primary tumor detection. The incorporation of both imaging‐informed EUA and, in selected cases, TORS‐TE/TBM in the diagnostic work‐up further improves the likelihood of detecting the primary tumor. A multimodal diagnostic approach, incorporating expert multi‐reader evaluation of imaging, improves primary tumor detection, contributing to better patient outcomes and more personalized treatment strategies.

## Funding

The authors have nothing to report.

## Ethics Statement

The Medical Ethics Review Committee of Amsterdam UMC approved this study with a waiver of informed consent (IRB00002991, FWA00017598).

## Conflicts of Interest

The authors declare no conflicts of interest.

## Data Availability

The data that support the findings of this study are available on request from the corresponding author. The data are not publicly available due to privacy or ethical restrictions.

## References

[1] A. Barsouk , J. S. Aluru , P. Rawla , K. Saginala , and A. Barsouk , “Epidemiology, Risk Factors, and Prevention of Head and Neck Squamous Cell Carcinoma,” Medical Science 11 (2023): 42. 10.3390/medsci11020042 PMC10304137 37367741

[2] K. Motz , J. R. Qualliotine , E. Rettig , J. D. Richmon , D. W. Eisele , and C. Fakhry , “Changes in Unknown Primary Squamous Cell Carcinoma of the Head and Neck at Initial Presentation in the Era of Human Papillomavirus,” JAMA Otolaryngology. Head & Neck Surgery 142 (2016): 223–228. 26769661 10.1001/jamaoto.2015.3228

[3] T. J. Galloway and J. A. Ridge , “Management of Squamous Cancer Metastatic to Cervical Nodes With an Unknown Primary Site,” Journal of Clinical Oncology 33 (2015): 3328–3337. 26351351 10.1200/JCO.2015.61.0063

[4] F. dos Santos Menezes , G. A. Fernandes , J. L. F. Antunes , L. L. Villa , and T. N. Toporcov , “Global Incidence Trends in Head and Neck Cancer for HPV‐Related And‐Unrelated Subsites: A Systematic Review of Population‐Based Studies,” Oral Oncology 115 (2021): 105177. 33561611 10.1016/j.oraloncology.2020.105177

[5] E. Rassy , P. Nicolai , and N. Pavlidis , “Comprehensive Management of HPV‐Related Squamous Cell Carcinoma of the Head and Neck of Unknown Primary,” Head & Neck 41 (2019): 3700–3711. 31301162 10.1002/hed.25858

[6] M. H. H. Larsen , H. I. Channir , and C. von Buchwald , “Human Papillomavirus and Squamous Cell Carcinoma of Unknown Primary in the Head and Neck Region: A Comprehensive Review on Clinical Implications,” Viruses 13 (2021): 1297. 34372502 10.3390/v13071297 PMC8310239

[7] C. Demiroz , J. M. Vainshtein , G. V. Koukourakis , et al., “Head and Neck Squamous Cell Carcinoma of Unknown Primary: Neck Dissection and Radiotherapy or Definitive Radiotherapy,” Head & Neck 36 (2014): 1589–1595. 23996575 10.1002/hed.23479 PMC4241546

[8] R. M. Martens , R. Stappen , T. Koopman , et al., “The Additional Value of Ultrafast DCE‐MRI to DWI‐MRI and 18F‐FDG‐PET to Detect Occult Primary Head and Neck Squamous Cell Carcinoma,” Cancers 12 (2020): 2826. 33007978 10.3390/cancers12102826 PMC7600235

[9] E. Maghami , N. Ismaila , A. Alvarez , et al., “Diagnosis and Management of Squamous Cell Carcinoma of Unknown Primary in the Head and Neck: ASCO Guideline,” Journal of Clinical Oncology 38 (2020): 2570–2596. 32324430 10.1200/JCO.20.00275

[10] L. M. Keller , T. J. Galloway , T. Holdbrook , et al., “p16 Status, Pathologic and Clinical Characteristics, Biomolecular Signature, and Long‐Term Outcomes in Head and Neck Squamous Cell Carcinomas of Unknown Primary,” Head & Neck 36 (2014): 1677–1684. 24115269 10.1002/hed.23514 PMC3972378

[11] L. Schroeder , M. Pring , K. Ingarfield , et al., “HPV Driven Squamous Cell Head and Neck Cancer of Unknown Primary Is Likely to Be HPV Driven Squamous Cell Oropharyngeal Cancer,” Oral Oncology 107 (2020): 104721. 32361566 10.1016/j.oraloncology.2020.104721

[12] M. Filauro , A. Paderno , P. Perotti , et al., “Role of Narrow‐Band Imaging in Detection of Head and Neck Unknown Primary Squamous Cell Carcinoma,” Laryngoscope 128 (2018): 2060–2066. 29392723 10.1002/lary.27098

[13] M. A. Pynnonen , M. B. Gillespie , B. Roman , et al., “Clinical Practice Guideline: Evaluation of the Neck Mass in Adults,” Otolaryngology and Head and Neck Surgery 157 (2017): S1–S30. 10.1177/0194599817722550 28891406

[14] F. J. Civantos , J. B. Vermorken , J. P. Shah , et al., “Metastatic Squamous Cell Carcinoma to the Cervical Lymph Nodes From an Unknown Primary Cancer: Management in the HPV Era,” Frontiers in Oncology 10 (2020): 593164. 33244460 10.3389/fonc.2020.593164 PMC7685177

[15] R. D. Chernock and J. S. Lewis , “Approach to Metastatic Carcinoma of Unknown Primary in the Head and Neck: Squamous Cell Carcinoma and Beyond,” Head and Neck Pathology 9 (2015): 6–15. 25804376 10.1007/s12105-015-0616-2 PMC4382479

[16] J. F. Ryan , K. M. Motz , L. M. Rooper , et al., “The Impact of a Stepwise Approach to Primary Tumor Detection in Squamous Cell Carcinoma of the Neck With Unknown Primary,” Laryngoscope 129 (2019): 1610–1616. 30565698 10.1002/lary.27625

[17] P. Golusinski , P. Di Maio , B. Pehlivan , et al., “Evidence for the Approach to the Diagnostic Evaluation of Squamous Cell Carcinoma Occult Primary Tumors of the Head and Neck,” Oral Oncology 88 (2019): 145–152. 30616785 10.1016/j.oraloncology.2018.11.020

[18] C. K. Sudoko , M. A. Polacco , B. J. Gosselin , and J. A. Paydarfar , “Diagnostic Value of Lingual Tonsillectomy in Unknown Primary Head and Neck Carcinoma Identification After a Negative Clinical Workup and Positron Emission Tomography‐Computed Tomography,” Frontiers in Oncology 8 (2018): 118. 29732318 10.3389/fonc.2018.00118 PMC5919999

[19] S. van Weert , J. A. Rijken , F. Plantone , et al., “A Systematic Review on Transoral Robotic Surgery (TORS) for Carcinoma of Unknown Primary Origin: Has Tongue Base Mucosectomy Become Indispensable?,” Clinical Otolaryngology 45 (2020): 732–738. 32369264 10.1111/coa.13565 PMC7496155

[20] A. De Virgilio , A. Costantino , D. Rizzo , et al., “Do We Have Enough Evidence to Specifically Recommend Transoral Robotic Surgery in HPV‐Driven Oropharyngeal Cancer? A Systematic Review,” Pathogens 12 (2023): 160. 36839432 10.3390/pathogens12020160 PMC9959572

[21] K. K. Gupta , H. Khan , Z. Mughal , M. De , N. Sharma , and G. Garas , “Primary Tumour Detection in Carcinoma of Unknown Primary With Transoral Robotic Surgery (TORS) Tongue Base Mucosectomy: A Meta‐Analysis,” Annals of Surgical Oncology 31 (2024): 6065–6076. 38980583 10.1245/s10434-024-15758-z

[22] M. Lanzer , S. Bachna‐Rotter , M. Graupp , et al., “Unknown Primary of the Head and Neck: A Long‐Term Follow‐Up,” Journal of Cranio‐Maxillofacial Surgery 43 (2015): 574–579. 25841309 10.1016/j.jcms.2015.03.004

[23] G. Madani , Z. Arain , and Z. Awad , “The Radiological Unknown Primary of the Head and Neck: Recommendations for Imaging Strategies Based on a Systematic Review,” Clinical Otolaryngology 49 (2024): 16–28. 37846889 10.1111/coa.14111

[24] J. C. Hardman , J. Constable , A. Williamson , et al., “Investigations for Suspected Head and Neck Squamous Cell Carcinoma of Unknown Primary (HNSCCUP): A National Cohort Study,” Clinical Otolaryngology 50 (2025): 462–473. 39779315 10.1111/coa.14272

[25] J. R. Lee , J. S. Kim , J. L. Roh , et al., “Detection of Occult Primary Tumors in Patients With Cervical Metastases of Unknown Primary Tumors: Comparison of (18)F FDG PET/CT With Contrast‐Enhanced CT or CT/MR Imaging‐Prospective Study,” Radiology 274 (2015): 764–771. 25405771 10.1148/radiol.14141073

[26] M. Godeny , Z. Lengyel , G. Polony , et al., “Impact of 3T Multiparametric MRI and FDG‐PET‐CT in the Evaluation of Occult Primary Cancer With Cervical Node Metastasis,” Cancer Imaging 16 (2016): 38. 27814768 10.1186/s40644-016-0097-x PMC5096285

[27] D. P. Noij , R. M. Martens , B. Zwezerijnen , et al., “Diagnostic Value of Diffusion‐Weighted Imaging and 18F‐FDG‐PET/CT for the Detection of Unknown Primary Head and Neck Cancer in Patients Presenting With Cervical Metastasis,” European Journal of Radiology 107 (2018): 20–25. 30292267 10.1016/j.ejrad.2018.08.009

[28] S. Van Weert , J.‐J. Hendrickx , and C. R. Leemans , “Carcinoma of Unknown Primary: Diagnostics and the Potential of Transoral Surgery,” in Critical Issues in Head and Neck Oncology: Key Concepts From the Eighth THNO Meeting (Springer International Publishing, 2023), 179–197.

[29] M. W. Chan , E. Yu , E. Bartlett , et al., “Morphologic and Topographic Radiologic Features of Human Papillomavirus‐Related and ‐Unrelated Oropharyngeal Carcinoma,” Head & Neck 39 (2017): 1524–1534. 28580605 10.1002/hed.24764

[30] M. Ravanelli , A. Grammatica , E. Tononcelli , et al., “Correlation Between Human Papillomavirus Status and Quantitative MR Imaging Parameters Including Diffusion‐Weighted Imaging and Texture Features in Oropharyngeal Carcinoma,” AJNR. American Journal of Neuroradiology 39 (2018): 1878–1883. 30213805 10.3174/ajnr.A5792 PMC7410748

[31] A. K. Tahari , K. C. Alluri , H. Quon , W. Koch , R. L. Wahl , and R. M. Subramaniam , “FDG PET/CT Imaging of Oropharyngeal Squamous Cell Carcinoma: Characteristics of Human Papillomavirus‐Positive and ‐Negative Tumors,” Clinical Nuclear Medicine 39 (2014): 225–231. 24152652 10.1097/RLU.0000000000000255 PMC4074504

[32] M. S. Hofman and R. J. Hicks , “How We Read Oncologic FDG PET/CT,” Cancer Imaging 16 (2016): 1–14. 27756360 10.1186/s40644-016-0091-3 PMC5067887

[33] M. Albertson , S. Chandra , Z. Sayed , and C. Johnson , “PET/CT Evaluation of Head and Neck Cancer of Unknown Primary,” in Seminars in Ultrasound, CT and MRI, vol. 40 (Elsevier, 2019), 414–423. 10.1053/j.sult.2019.07.005 31635768

[34] M. Faisal , N.‐S. Le , S. Grasl , et al., “Carcinoma of Unknown Primary (CUP) Versus CUP Turned to Primary Carcinoma of the Head and Neck—An Analysis of Diagnostic Methods and the Impact of Primary Tumor on Clinical Outcome,” Diagnostics 12 (2022): 894. 35453942 10.3390/diagnostics12040894 PMC9032826

[35] M. Y. Lee , N. Fowler , D. Adelstein , S. Koyfman , B. Prendes , and B. B. Burkey , “Detection and Oncologic Outcomes of Head and Neck Squamous Cell Carcinoma of Unknown Primary Origin,” Anticancer Research 40 (2020): 4207–4214. 32727746 10.21873/anticanres.14421

[36] M. W. Kubik , H. I. Channir , N. Rubek , et al., “TORS Base‐Of‐Tongue Mucosectomy in Human Papilloma Virus‐Negative Carcinoma of Unknown Primary,” Laryngoscope 131 (2021): 78–81. 32239774 10.1002/lary.28617

[37] D. Wang , T. Zou , T. Gao , et al., “Diagnosis and Prognosis of Different Methods of Tongue Base Mucosectomy for Occult Head and Neck Cancer: A Systematic Review and Meta‐Analysis,” Medicine 103 (2024): e40250. 39560574 10.1097/MD.0000000000040250 PMC11576005

[38] F. A. van der Scheer , F. Jansen , S. E. J. Eerenstein , et al., “Swallowing Outcomes After Transoral Robotic Surgery and Adjuvant Treatment in Unknown Primary,” Oral Diseases 30 (2024): 4830–4837. 38988121 10.1111/odi.15063 PMC11610685

[39] S. Farooq , S. Khandavilli , J. Dretzke , et al., “Transoral Tongue Base Mucosectomy for the Identification of the Primary Site in the Work‐Up of Cancers of Unknown Origin: Systematic Review and Meta‐Analysis,” Oral Oncology 91 (2019): 97–106. 30926070 10.1016/j.oraloncology.2019.02.018

[40] S. Winter , E. Ofo , D. Meikle , et al., “Trans‐Oral Robotic Assisted Tongue Base Mucosectomy for Investigation of Cancer of Unknown Primary in the Head and Neck Region. The UK Experience,” Clinical Otolaryngology 42 (2017): 1247–1251. 28258624 10.1111/coa.12860

[41] S. A. Patel , A. Parvathaneni , U. Parvathaneni , et al., “Post‐Operative Therapy Following Transoral Robotic Surgery for Unknown Primary Cancers of the Head and Neck,” Oral Oncology 72 (2017): 150–156. 28797451 10.1016/j.oraloncology.2017.07.019

[42] H. I. Channir , N. Rubek , H. U. Nielsen , et al., “Transoral Robotic Surgery for the Management of Head and Neck Squamous Cell Carcinoma of Unknown Primary,” Acta Oto‐Laryngologica 135 (2015): 1051–1057. 26073750 10.3109/00016489.2015.1052983

[43] C. M. Yver , D. Shimunov , G. S. Weinstein , et al., “Oncologic and Survival Outcomes for Resectable Locally‐Advanced HPV‐Related Oropharyngeal Cancer Treated With Transoral Robotic Surgery,” Oral Oncology 118 (2021): 105307. 33932874 10.1016/j.oraloncology.2021.105307

[44] F. C. A. Xavier , J. C. Silva , C. O. Rodini , and M. Rodrigues , “Mechanisms of Immune Evasion by Head and Neck Cancer Stem Cells,” Frontiers in Oral Health 3 (2022): 957310. 35982868 10.3389/froh.2022.957310 PMC9378780

[45] K. L. Kostecki , M. Iida , B. E. Crossman , et al., “Immune Escape Strategies in Head and Neck Cancer: Evade, Resist, Inhibit, Recruit,” Cancers 16 (2024): 312. 38254801 10.3390/cancers16020312 PMC10814769

[46] T. Muijlwijk , D. Nijenhuis , S. H. Ganzevles , et al., “Comparative Analysis of Immune Infiltrates in Head and Neck Cancers Across Anatomical Sites,” Journal for Immunotherapy of Cancer 12 (2024): e007573. 38212122 10.1136/jitc-2023-007573 PMC10806653

[47] F. Bochen , H. Adisurya , S. Wemmert , et al., “Effect of 3q Oncogenes SEC62 and SOX2 on Lymphatic Metastasis and Clinical Outcome of Head and Neck Squamous Cell Carcinomas,” Oncotarget 8 (2017): 4922–4934. 28002801 10.18632/oncotarget.13986 PMC5354881

